# An Intronic *MBTPS2* Variant Results in a Splicing Defect in Horses with Brindle Coat Texture

**DOI:** 10.1534/g3.116.032433

**Published:** 2016-07-22

**Authors:** Leonardo Murgiano, Dominik P. Waluk, Rachel Towers, Natalie Wiedemar, Joëlle Dietrich, Vidhya Jagannathan, Michaela Drögemüller, Pierre Balmer, Tom Druet, Arnaud Galichet, M. Cecilia Penedo, Eliane J. Müller, Petra Roosje, Monika M. Welle, Tosso Leeb

**Affiliations:** * Institute of Genetics, Vetsuisse Faculty, University of Bern, 3001 Switzerland; † Dermfocus, Vetsuisse Faculty, University of Bern, 3001 Switzerland; ‡ Unit of Animal Genomics, GIGA-R and Faculty of Veterinary Medicine, University of Liège, 4000 Belgium; § Department of Clinical Research, Molecular Dermatology and Stem Cell Research, University of Bern, 3008 Switzerland; ** Institute of Medical Genetics, Cardiff University, CF14 4XN, UK; †† Division of Clinical Dermatology, Department of Clinical Veterinary Medicine, Vetsuisse Faculty, University of Bern, 3001 Switzerland; ‡‡ Veterinary Genetics Laboratory, School of Veterinary Medicine, University of California, Davis, California 95617-1102; §§ Institute of Animal Pathology, Vetsuisse Faculty, University of Bern, 3001 Switzerland; *** Clinic for Dermatology, Inselspital, Bern University Hospital, 3010 Switzerland

**Keywords:** *Equus Caballus*, X-chromosome, dermatology, hair, lines of Blaschko

## Abstract

We investigated a family of horses exhibiting irregular vertical stripes in their hair coat texture along the neck, back, hindquarters, and upper legs. This phenotype is termed “brindle” by horse breeders. We propose the term “brindle 1 (BR1)” for this specific form of brindle. In some BR1 horses, the stripes were also differentially pigmented. Pedigree analyses were suggestive of a monogenic X-chromosomal semidominant mode of inheritance. Haplotype analyses identified a 5 Mb candidate region on chromosome X. Whole genome sequencing of four BR1 and 60 nonbrindle horses identified 61 private variants in the critical interval, none of them located in an exon of an annotated gene. However, one of the private variants was close to an exon/intron boundary in intron 10 of the *MBTPS2* gene encoding the membrane bound transcription factor peptidase, site 2 (c.1437+4T>C). Different coding variants in this gene lead to three related genodermatoses in human patients. We therefore analyzed *MBTPS2* transcripts in skin, and identified an aberrant transcript in a BR1 horse, which lacked the entire exon 10 and parts of exon 11. The *MBTPS2*:c1437+4T>C variant showed perfect cosegregation with the brindle phenotype in the investigated family, and was absent from 457 control horses of diverse breeds. Altogether, our genetic data, and previous knowledge on *MBTPS2* function in the skin, suggest that the identified *MBTPS2* intronic variant leads to partial exon skipping, and causes the BR1 phenotype in horses.

In heterozygous females, X-linked skin conditions frequently result in a characteristic patterning of the skin, following the so-called lines of Blaschko (Happle 2006; Molho-Pessach and Schaffer 2011; Happle 2016). The Blaschko lines describe stripes of affected skin follow specific patterns along lines of the body. These lines, S-shaped on the side of the body, and V-shaped on the back, follow the trajectory of cell migration and proliferation during embryogenesis. These characteristic patterns are the result of X-chromosome inactivation (also called lyonization). In heterozygous females, the lesions are restricted to skin areas that derive from cell clones, in which the wild-type X-chromosome has become inactivated, and only the mutant X-chromosome is expressed. X-chromosome inactivation thus leads to functional mosaicism, and explains different patterns that do not correspond to the nervous, muscular, or lymphatic system (Happle 2006; Sun and Tsao 2008). Examples of human X-linked genodermatoses showing this phenomenon are incontinentia pigmenti, focal dermal hypoplasia, and hypohidrotic ectodermal dysplasia (Smahi *et al.* 2000; Grzeschik *et al.* 2007; X. Wang *et al.* 2007; Kere *et al.* 1996).

The investigation of spontaneous domestic animal mutants displaying skin phenotypes can yield new insights into the functions of genes during epidermal development or in the regulation of skin homeostasis (Drögemüller *et al.* 2008, 2014; Jagannathan *et al.* 2013). We previously reported X-linked skin conditions that lead to striped patterns, including the streaked hairlessness in Italian Pezzata Rossa cattle, which is caused by a *TSR2* splice site variant (Murgiano *et al.* 2015), and incontinentia pigmenti in horses, which is caused by an *IKBKG* nonsense variant (Towers *et al.* 2013). Horses with incontinentia pigmenti display pruritic, exudative skin lesions soon after birth. These develop into wart-like lesions and areas of alopecia. Affected horses also have streaks of darker and lighter coat coloration from birth. These cutaneous manifestations in horses with incontinentia pigmenti follow the lines of Blaschko (Towers *et al.* 2013). In the horse family segregating for incontinentia pigmenti, an independent, but closely related phenotype with a striped coat texture pattern has long been recognized by breeders, and is termed brindle. The term brindle has been used for similar phenotypes in different breeds, and may in fact refer to horses with different genetic alterations, including rare spontaneous chimeras (Sponenberg 2009). Due to the ambiguities associated with the term brindle, we will use the term brindle 1 (BR1) for the specific brindle pattern observed in the investigated family.

In contrast to horses with incontinentia pigmenti, BR1 horses do not show any hoof or teeth abnormalities. The aim of the present study was to characterize the genetics underlying the BR1 phenotype. As this phenotype has never been fully described in the scientific literature, we also present a preliminary qualitative characterization of the BR1 phenotype.

## Materials and Methods

### Samples and phenotypes

Three experienced breeders submitted hair or EDTA blood samples from 39 closely related horses segregating for both incontinentia pigmenti (Towers *et al.* 2013) and the BR1 phenotype for this study. Most of these horses had an American Quarter Horse or American Paint Horse registration, but some were also registered as warmblood horses. The phenotype classification into BR1 and control horses was based on the breeders’ reports.

We additionally used samples from 457 unrelated horses from diverse breeds not suspected to have the BR1 phenotype, which had been collected in the course of other projects at the Institute of Genetics of the University of Bern. We isolated genomic DNA from hair roots or EDTA blood samples according to standard procedures.

### Hair samples and hair morphology analyses

**Figure 1:**
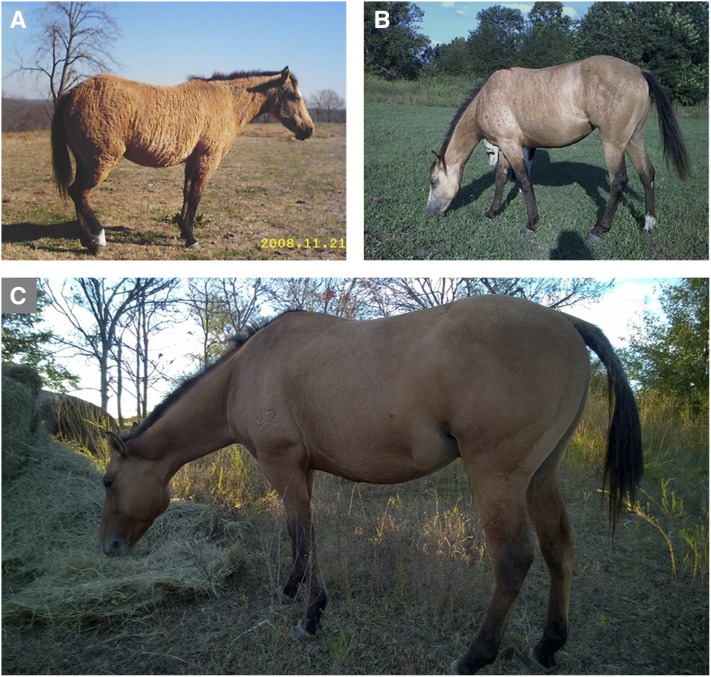
Hair coat phenotype of BR1 horses. Vertical stripes and irregular coat are macroscopically visible in the (A) winter and (B) summer coat of the same BR1 mare (UKH23). (C) The stallion UKH38 born out of a BR1 mare did not show the pronounced striped pattern, but had a very sparse mane and tail.

**Figure 2:**
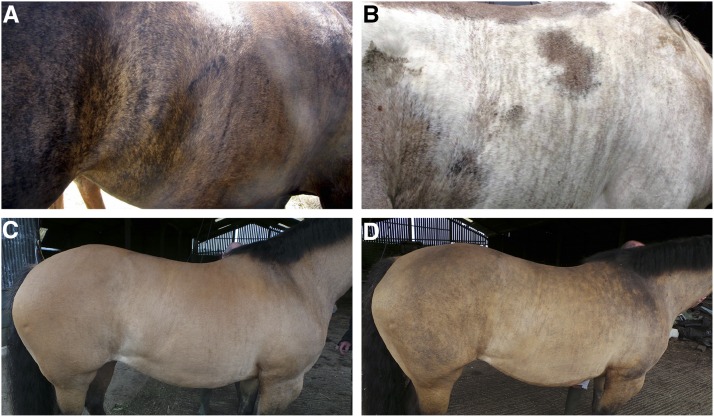
Pigmentation phenotype of BR1 mares. (A) Pronounced alternating stripes of eumelanin and pheomelanin in a horse with solid coat color (UKH14). (B) The horse UKH11 had the frame overo spotting phenotype caused by the *EDNRB*:c.354_355delinsAG variant, and a bay base color. This horse showed fine lines of pigmented skin in areas that would be devoid of melanocytes and pigmentation in nonbrindle horses with the frame-overo spotting pattern. (C) The BR1 mare UKH004 showed only a very subtle pattern of striped pigmentation on her coat. (D) The same mare UKH004 showed a much more pronounced pattern of irregular pigmentation in her skin after clipping the hair. These photos illustrate different types of streaky pigmentation seen in some BR1 horses.

One breeder submitted hairs of lesional and nonlesional areas from four female BR1 horses (UKH11, UKH15, UKH21, and UKH23) in separately labeled bags for macroscopic and microscopic examination. Nonlesional was defined as hair from the base colored, or normal textured, coat and “lesional” was defined as hair from the phenotypically different coat stripes (abnormal texture and striping as seen in Figure 1 and Figure 2). Samples were coded by one investigator (P. R.). Another investigator (P. B.) analyzed the macroscopic and microscopic appearance without knowing whether they came from lesional or nonlesional areas. The following parameters were quantitatively assessed in three hairs from each sample: length of the hairs, surface area of the roots, diameter of the hair shafts at four different positions (25% of shaft length, 50% of shaft length, 75% of shaft length, tip of the hair, where the medulla ends), and diameter of the root. Student’s *t*-tests with a significance threshold of *P* = 0.05 were performed for statistical analysis. In addition to the quantitative parameters, the curvature of the hairs shafts was also assessed qualitatively.

### Skin biopsies and histopathological examination

Two 6 mm punch biopsies were obtained from the skin of a BR1 horse (UKH04) after local anesthesia with 2% lidocaine. The biopsies were collected from the lesional streaks and from normal (nonlesional) areas. Biopsies were fixed in 4% buffered formalin, processed routinely, and stained with hematoxylin and eosin prior to histopathological examination. Two further biopsies from comparable sites on the same horse were submerged in RNAlater solution for subsequent RNA isolation.

### Genotyping

Genotyping of 22 BR1 and 14 control horses was performed by Geneseek/Neogen using the illumina Equine SNP70 chip containing 73,774 evenly distributed SNPs.

### Haplotype reconstruction

We used LINKPHASE3 (Druet and Georges 2015) to reconstruct haplotypes in the genotyped pedigree based on Mendelian segregation rules and linkage information. Detailed information on the methodology and modifications required to analyze the X-chromosome are described in Supplemental Material, File S1.

### Whole genome resequencing, SNP and short indel calling

We prepared PCR-free genomic fragment libraries with 350 bp insert size and collected roughly 20× coverage data on an Illumina HiSeq2500 or HiSeq3000 instrument (2 × 100, 2 × 125, or 2 × 150 bp). Sequence reads were mapped to the horse reference genome, and aligned using Burrows-Wheeler Aligner (BWA) version 0.5.9-r16 (Li and Durbin 2009) with default settings. The SAM file generated by BWA was then converted to BAM, and the reads sorted by chromosome using samtools (Li 2011). PCR duplicates were marked using Picard tools (http://sourceforge.net/projects/picard/). We used the Genome Analysis Tool Kit (GATK version 2.4.9, 50) to perform local realignment, and to produce a cleaned BAM file. Variant calls were then made with the Unified Genotyper module of GATK (McKenna *et al.* 2010). The variant data for each sample was obtained in variant call format (version 4.0) as raw calls for all samples, and sites flagged using the variant filtration module of GATK. Variant filtration was performed, following best practice documentation of GATK version 4. The snpEFF software (Cingolani *et al.* 2012) together with the EquCab 2.0 annotation was used to predict the functional effects of detected variants.

### Structural variant calling

We used the Delly package (Rausch *et al.* 2012) to look for larger deletions in the sequenced family trio, using daughter and mother as case and the father as control. Delly uses paired-ends and split-reads to sensitively and accurately delineate genomic rearrangements throughout the genome. Delly was run four times; each time, the command line contained either the DEL, DUP, INV, or TRA command after the –t command that specifies the request to look for deletions, tandem duplications, inversions, and translocations, respectively.

To identify whether copy number variation was associated with the BR1 phenotype, we used the software PennCNV (K. Wang *et al.* 2007). PennCNV uses a hidden Markov model based approach that takes into account signal intensity, allelic intensity ratio, distance between markers, and allele frequency to call CNVs. We built the input consisting into the PFB file, the intensity file and GCModel file from the data obtained from the manufacturer, using 22 animals present in one single SNP batch.

### Sanger sequencing

The associated *MBTPS2* variant was genotyped by resequencing of targeted PCR products (forward-primer: GCTACTCCAATTTTCAAGCGT, reverse-primer: TCCCTGCCACATCTTAACCT) using Sanger sequencing technology. PCR products were amplified with AmpliTaqGold360Mastermix (Life Technologies), and the products directly sequenced using the PCR primers on an ABI 3730 capillary sequencer (Life Technologies) after treatment with exonuclease I and shrimp alkaline phosphatase. Sequence data were analyzed using Sequencher 5.1 (GeneCodes).

### In silico splice site analysis

We used the “Splice-Site Analyzer Tool”, for a computational analysis of the strength of the wildtype and mutant 5′-splice sites of the equine *MBTPS2* gene. This program is freely available on the internet (http://ibis.tau.ac.il/ssat/SpliceSiteFrame.htm), and analyzes the free energy for the binding of the primary transcript to the U1 RNA of the spliceosome. The program is based on a dataset of 50,493 homologous human-mouse internal exons (Carmel *et al.* 2004).

### RT-PCR

The RNA was extracted from skin tissues using the RNeasy mini kit (Qiagen). The tissue was first finely crushed in TRIZOL (Ambion) using mechanical means, chloroform was then added and the RNA was separated by centrifugation. The RNA was cleared of genomic DNA contamination using the Quantitect Reverse Transcription Kit (Qiagen). The same kit was used to synthetize cDNA, as described by the manufacturer. An RT-PCR was carried out using primer MBTPS2_Ex8_F, ACAAACGGCTAGATGGTTCG, located in exon 8 and primer MBTPS2_Ex11_R, ATCCACTGTCCATCCAAAGC, located in exon 11 of the *MBTPS2* gene. The products were analyzed on a Fragment Analyzer capillary gel electrophoresis instrument (Advanced Analytical). To estimate the proportion of the aberrant *MBTPS2* transcript, we divided the peak area of the smaller *MBTPS2* band by the sum of the peak areas of both *MBTPS2* bands. The sequence of the obtained PCR product was confirmed by Sanger sequencing as described above. All numbering within the equine *MBTPS2* gene was done with respect to the reference transcript, GenBank accession XM_005614038.2.

### Data availability

Figure S1 shows the Sanger sequence data of *MBTPS2* transcripts. File S1 describes the detailed methodology for the haplotype analyses. Table S1 gives the gene annotation of the BR1 critical interval on the X chromosome as taken from NCBI MapViewer and the EquCab 2 assembly. Table S2 contains the complete list of 61 BR1-associated genetic variants in the critical interval. Table S3 provides a list of the numbers and breeds of the 457 control horses used for the genetic association study. The whole genome sequencing data from 64 horses were deposited with the European Nucleotide Archive. The accession numbers are listed in Table S4.

## Results

### Qualitative phenotype description

We previously reported a family of horses segregating incontinentia pigmenti characterized by consecutive characteristic stages (cutaneous pruritic, exudative lesions at birth, later on wart-like lesions, and additional tooth, hoof, and ocular anomalies; Towers *et al.* 2013). In the same horse family, breeders observed a milder phenotype that is restricted to an irregular striped texture and color pattern of the hair coat (Figure 1). The stripes generally formed a vertical pattern from the back to the sides of the animal. Breeders termed this phenotype “brindle” and we suggest brindle 1 or BR1 for this specific form of brindle. The observed BR1 phenotypes are quite variable in intensity between horses, and the phenotype also changes with season (summer *vs.* winter hair coat). The winter coat of BR1 horses often adopted a “moth eaten” appearance (Figure 1A). Some BR1 horses had sparse mane and tail hair. The BR1 phenotype occurred in horses with different base coat colors. In addition to the coat structure changes, some brindle horses also displayed vertical stripes of different pigmentation (Figure 2).

**Figure 3:**
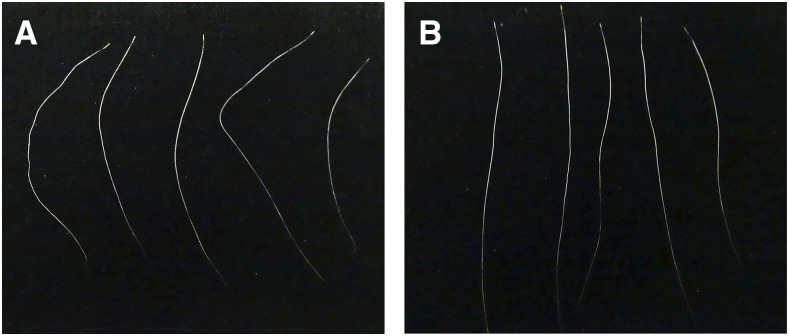
Macroscopic hair phenotype. The hairs from the lesional stripes (A) are more unruly compared to hairs from a nonlesional area of the same BR1 horse (B). Hairs were sampled during winter.

Macroscopic examination of the hairs revealed that hairs from the streaks were less straight, or were unruly, compared to the hairs from the normally pigmented and textured coat (Figure 3). No consistent difference in hair length, pigmentation, or diameter could be observed between the investigated lesional and nonlesional samples. On microscopic evaluation, we did not find any significant differences between the lesional and nonlesional hairs.

A histopathological examination in one brindle mare did not detect any difference between lesional and nonlesional skin areas. As expected at the end of winter, the vast majority of hair follicles were in telogen, or early anagen, and no difference concerning the pigmentation or morphology of the hair shafts, or the differentiation of hair follicles, was histologically evident between biopsies taken from two different locations.

### An X-linked mechanism of inheritance

**Figure 4:**
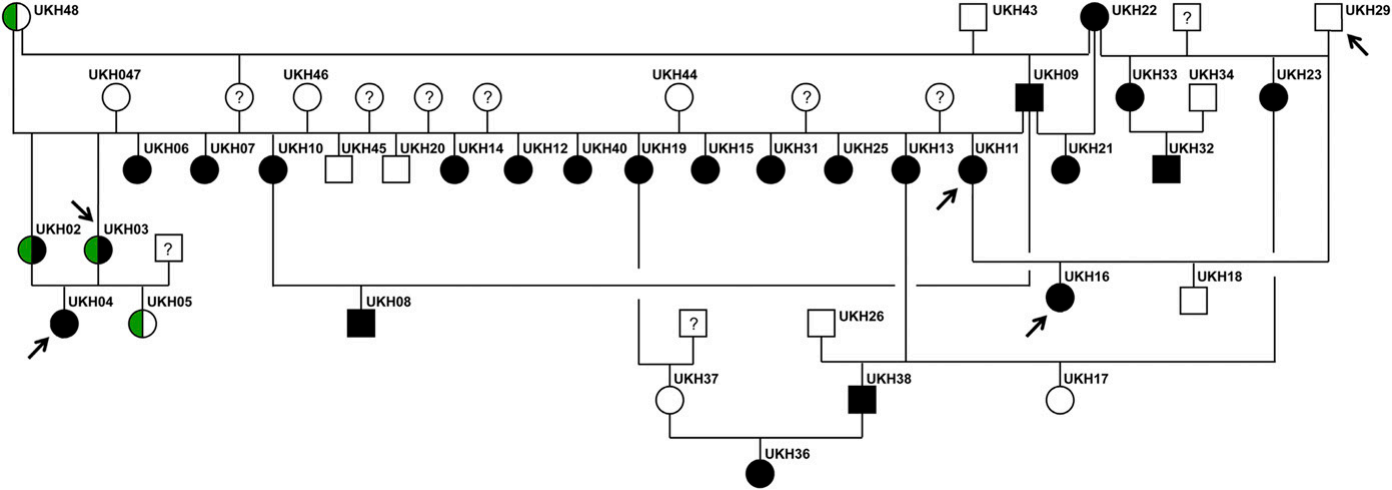
Family tree of BR1 horses. Males are represented by squares, females by circles. Animals showing the BR1 phenotype are indicated with filled black symbols. Affected males do not show the striped coat texture pattern, but they have a sparse mane and tail. Symbols filled in half with green color indicate mares affected by incontinentia pigmenti, which was confirmed by genotyping the *IKBKG*:c.C>T variant. Arrows indicate the animals for which whole genome sequences were produced. The mare UKH22, born in 1985, represents the putative founder of the BR1 phenotype.

All BR1 horses investigated during this study are descendants of a single Quarter Horse mare UKH22, born in 1985. We compiled a pedigree of the horses sampled for this study (Figure 4). Most notably, all daughters of the stallion UKH09 were reported to exhibit the BR1 phenotype, whereas all of his sons produced by matings with wildtype mares were normal. This stallion did not show the typical striped pattern of the hair coat, but had a sparse mane and tail. Based on the pedigree and the phenotype of the stallion UKH09, we hypothesized that BR1 is inherited as a monogenic X-chromosomal semidominant trait. According to this hypothesis, the “typical” BR1 phenotype with a striped coat texture pattern can only be seen in heterozygous females, whereas hemizygous mutant males and homozygous mutant females will primarily show a sparse mane and tail, but no stripes.

### Mapping of a candidate region

To localize the BR1 gene, we genotyped 36 horses from the sampled family on the equine illumina 70 k SNP chip. We reconstructed the haplotypes in the genotyped family and searched for the presence of shared haplotypes among the BR1 horses. The likelihood of a shared haplotype among the BR1 horses was null for all marker positions except for a 5 Mb segment on chromosome X spanning from positions 13,601,933 to 18,711,357 (EquCab2 assembly; markers BIEC2-1111129 - BIEC2-1112988). Only for that segment, the likelihood of a shared haplotype was higher than 0.99. Thus the haplotype analysis confirmed our hypothesis of an X-linked mode of inheritance. There were 41 annotated genes in the critical interval (Table S1).

### Identification of the causative variant

We sequenced the genomes of a trio consisting of a BR1 female (UKH16) together with her BR1 mother (UKH11), and her nonaffected father (UKH029), as well as one additional BR1 horse (UKH04). From our previous project on incontinentia pigmenti, we additionally had the genome sequence of an incontinentia pigmenti horse from the same family, which also carried the BR1 haplotype (UKH03). Finally, we had whole genome sequence data from 59 additional horses from diverse breeds not suspected to have the BR1 phenotype, which had been obtained previously in the course of other projects. Thus, we had four female genomes predicted to be heterozygous at the BR1 locus, and 60 control genomes, which should be either hemizygous or homozygous wildtype at the causative variant. Together, these 64 horse genomes comprised > 62 million genetic variants.

Filtering for high-confidence heterozygous single nucleotide and small indel variants, private to the four BR1 cases, yielded 61 such variants in the critical interval on the X-chromosome. None of these 61 variants were located in an exon of an annotated gene (Table S2). A search for structural variants using the programs Delly and PennCNV did not identify any such variants in the critical interval.

**Figure 5:**
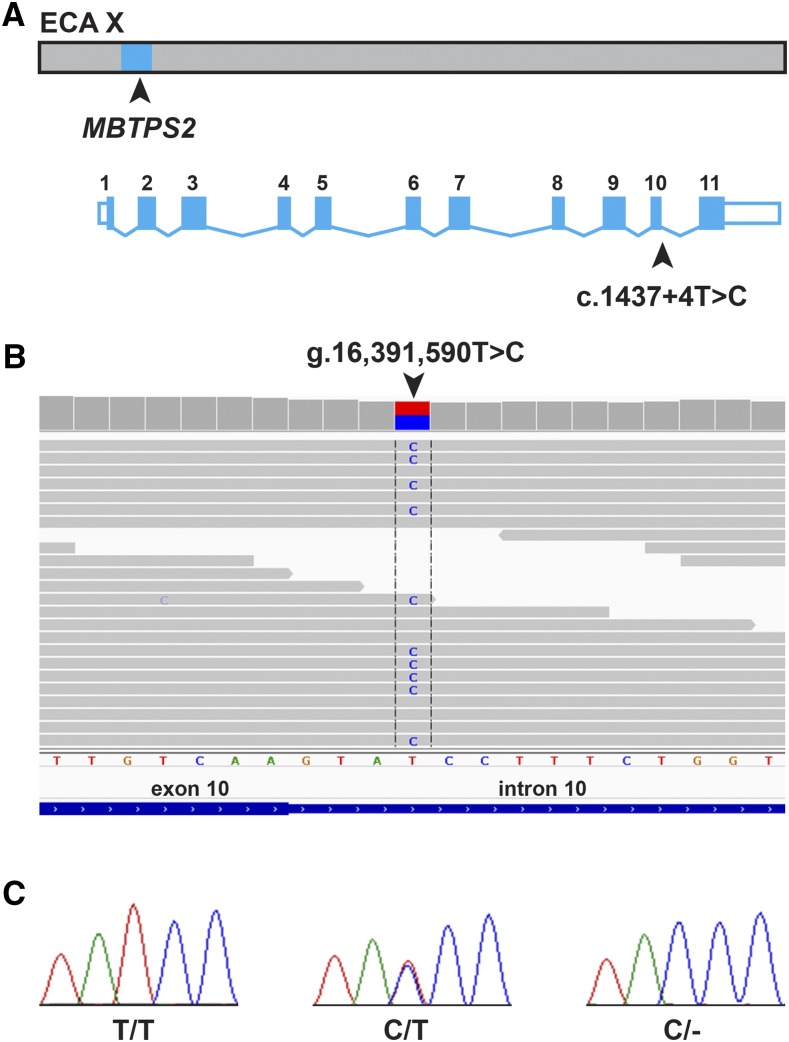
Genomic context of the *MBTPS2*:c.1437+4T>C variant. (A) The 5 Mb haplotype on the X-chromosome, which was shared by all BR1 horses, is shown in light blue. The *MBTPS2* genomic organization and the position of the variant are indicated (exons and introns are not drawn to scale). (B) Screenshot of the illumina sequence reads of a brindle mare indicating the presence of a heterozygous T>C variant. (C) Sanger electropherograms of a homozygous wildtype female, a heterozygous female, and a hemizygous mutant male.

Visual inspection of the 61 candidate variants for their potential functional relevance led us to focus our attention on a single nucleotide variant g.16,391,590T>C, which was located at position +4 of intron 10 of the *MBTPS2* gene (c.1437+4T>C). We confirmed the presence of this variant by Sanger sequencing (Figure 5).

### Genetic association of the MBTPS2:c.1437+4T>C variant

We genotyped the 39 available horses from the investigated family, and found perfect cosegregation of the variant with the BR1 phenotype. In this family, we observed 14 nonaffected horses, which were homozygous or hemizygous for the wildtype allele. We had 21 heterozygous females; 19 of these heterozygous females showed the BR1 phenotype, and, in the remaining two, the BR1 phenotype could not be seen due to the simultaneous presence of the more severe incontinentia pigmenti phenotype. Finally, we had four hemizygous mutant males with sparse mane and tail.

We further genotyped 457 control horses from 17 genetically diverse horse and pony breeds for the *MBTPS2*:c.1437+4T>C variant (Table S3). The mutant allele did not occur in any of these horses.

### An MBTPS2 splice defect

Due to its position next to an exon/intron boundary, we hypothesized that the *MBTPS2*:c.1437+4T>C variant might have a potential impact on the correct splicing of *MBTPS2* transcripts, and thereby cause the phenotype. An *in silico* analysis of the wildtype (CAAgta**t**cc) and mutant (CAAgta**c**cc) 5′-splice site sequences of intron 10 predicted a slightly reduced score for the binding of the mutant 5′-splice site to the U1 RNA of the spliceosome. Both sequences represented relatively weak 5′-splice sites.

**Figure 6:**
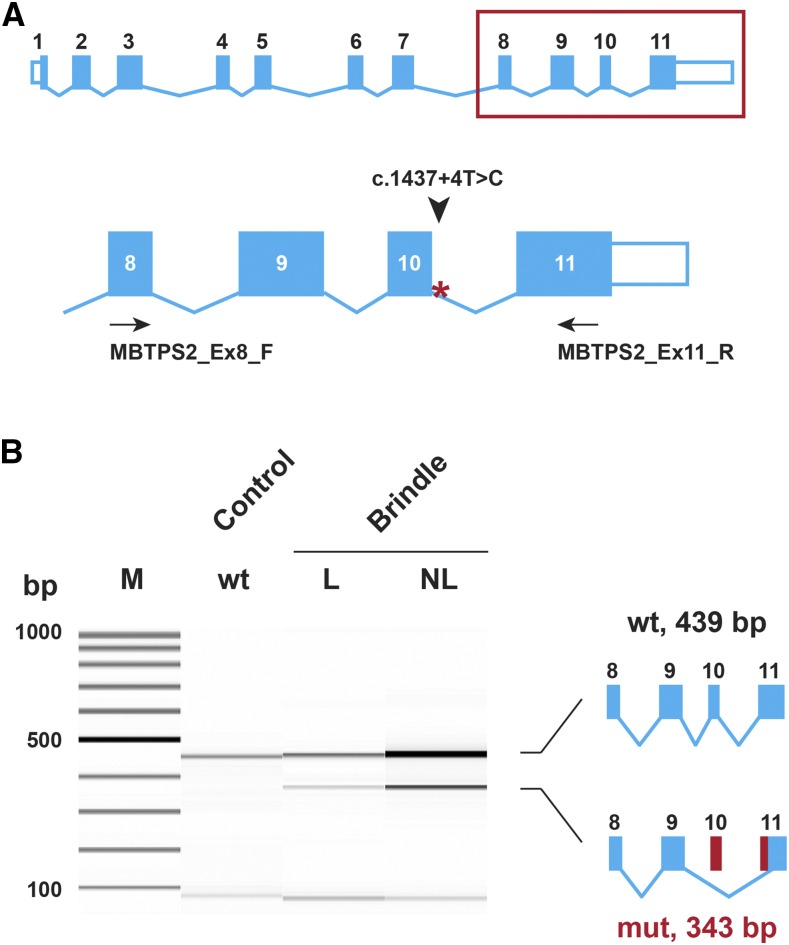
Experimental verification of the *MBTPS2* splice defect. (A) The *MBTPS2* transcript is shown. On the enlarged 3′-end of the transcript, the position of the primers used for RT-PCR is indicated. (B) RT-PCR was performed using skin RNA from a brindle and a control horse. The picture shows a Fragment Analyzer gel image of the experiment. In the control animal (wt), only the expected 439 bp band is visible. For the brindle horse, two RNA samples from lesional (L) and nonlesional (NL) skin were used. In both samples, an additional 343 bp band corresponding to a transcript lacking the entire exon 10 and parts of exon 11 can be seen. The variant designation on the transcript level thus is r.[=,1265_1360del]. The band at the bottom of all three lanes with RT-PCR products corresponds to molecules of < 50 nt in size, and probably consists of residual unused primers or primer dimers. The aberrant 343 bp band comprised 23% and 19% of the total transcripts in the lesional and nonlesional skin sample, respectively.

We performed an RT-PCR to experimentally test the consequences of the 5′-splice site variant. Primers located in exons 8 and 11 of the *MBTPS2* gene were used to amplify cDNA from the skin of a BR1 horse and an unrelated control. The BR1 horse expressed the wildtype transcript, but also a transcript that lacked 96 nucleotides (nt) consisting of the entire exon 10 and the first 20 nt of exon 11, confirming the existence of an aberrantly spliced *MBTPS2* transcript in the BR1 horse (Figure 6 and Figure S1). The identity of the RT-PCR bands was confirmed by direct Sanger sequencing. The proportion of the mutant transcript in the BR1 horse was roughly 20%, and we observed a minimally higher proportion of mutant transcript in the skin biopsy taken from a lesional skin area compared to the skin biopsy taken from a nonlesional skin area. However, as we had only two biopsies from one horse for this analysis, it is unclear whether this difference is significant. The mutant transcript contained an open reading frame lacking 32 codons, which encode parts of the third luminal and the entire sixth transmembrane domain of the MBTPS2 protein (p.422_453del).

## Discussion

In this study, we provide an initial and qualitative phenotypic description of one form of brindle in horses, termed “brindle 1 (BR1)”. This form of brindle is phenotypically quite variable, and some horses are predominantly characterized by coat texture changes, while, in others, the striped pigmentation pattern is the predominant feature. More comprehensive quantitative morphometric studies will be needed to fully characterize all changes in hair structure and pigmentation that can occur in BR1 horses. It will also be interesting to see whether hemizygous mutant males and homozygous mutant females will show some subtle alteration of their body hair in addition to their sparse manes and tails.

We mapped the causative locus for this form of brindle in horses to a 5 Mb segment on the X-chromosome. A whole genome sequencing approach identified a single nucleotide variant within the 5′-splice site of intron 10 of the *MBTPS2* gene. We have to caution that the equine reference genome assembly and annotation are not perfect, which impedes our variant detection sensitivity. For example, the horse genome reference contains two gap regions within the equine *MBTPS2* gene, one of them harboring exon 9, with 196 coding nt. Thus we cannot exclude the possibility that we missed one or several additional genetic variants in linkage disequilibrium with the 61 identified private variants. However, we can at least rule out exon 9 of the *MBTPS2* gene, as this exon was unchanged in the cDNA Sanger sequencing data from a BR1 horse.

The *MBTPS2*:c.1437+4T>C variant cosegregated perfectly with the phenotype in an extended horse family, and was absent from a large cohort of horses from diverse breeds. We further confirmed the expression of an additional aberrantly spliced *MBTPS2* transcript in the skin of a BR1 horse.

The *MBTPS2* gene encodes the membrane bound transcription factor peptidase, site 2, a zinc metalloprotease located in the membrane of the endoplasmic reticulum (ER) and Golgi apparatus. MBTPS2 cleaves sterol regulatory element binding proteins (SREBPs) within a transmembrane segment, releasing the amino-terminal domain of the SREBP from the membrane so that it can enter the nucleus and activate target genes encoding *e.g.*, the low density lipoprotein receptor and enzymes for cholesterol and fatty acid biosynthesis (Rawson *et al.* 1997; Zelenski *et al.* 1999; Kroos and Akiyama 2013).

Genetic variants in *MBTPS2* lead to three related genodermatoses in human patients: ichthyosis follicularis, atrichia, and photophobia (IFAP, OMIM #308205, Oeffner *et al.* 2009), Olmsted syndrome/palmoplantar keratoderma (OLMSX, OMIM #300918, Haghighi *et al.* 2013), and keratosis follicularis spinulosa decalvans (KFSDX, OMIM #308800, Aten *et al.* 2010). A detailed genotype–phenotype correlation for a large number of different human variants has been published (Bornholdt *et al.* 2013). It is thought that the complete absence of functional MBTPS2 is lethal. Depending on the specific genetic variant, male human patients carrying missense mutations in the *MBTPS2* gene show mostly quite severe phenotypes, sometimes with additional developmental aberrations and/or mental retardation in addition to the skin changes termed BRESEK/BRESHECK syndrome (Reish *et al.* 1997). Females carrying any of these *MBTPS2* gene variants in heterozygous state show skin changes of varying severity that follow the lines of Blaschko, or no visible phenotype at all. Male human IFAP patients with intronic *MBTPS2* variants affecting splicing were reported. Unfortunately, RNA of these patients was not available, and it is therefore unknown which proportions of wildtype and aberrant transcripts were expressed (Oeffner *et al.* 2011).

It is not fully elucidated how *MBTPS2* deficiency mechanistically causes the observed skin and hair changes in human patients. *MBTPS2* deficiency is thought to lead to changes in ER stress response and sterol homeostasis, including cholesterol biosynthesis. Several other defects in genes required for steroid and lipid metabolism are also associated with genodermatoses, such as, *e.g.*, ichthyoses (Feingold and Elias 2014).

Based on the fact that hair morphology is altered in both BR1 horses and human patients with *MBTPS2* variants, we propose that the observed splice defect is actually causative for the BR1 phenotype in horses. The BR1 phenotype is clearly much milder than the different phenotypes reported in human patients with coding *MBTPS2* variants. We speculate that this might be due to the specific equine variant and the high proportion of wildtype transcript, which is still expressed in the skin of mutant horses. It remains to be investigated whether the predicted truncated MBTPS2 protein is actually expressed and correctly localized in BR1 horses. In this case, it could potentially exert a dominant-negative function in cells that express both wildtype and mutant protein (gain of function). On the other hand, it is also possible that the truncated protein is not correctly processed, and is rapidly degraded. In this case, BR1 horses might be lacking sufficient amounts of functional MBTPS2 protein to maintain a completely normal coat appearance (loss of function).

The obtained data do not definitively prove the causality of the *MBTPS2*:c.1437+4T>C variant for the BR1 phenotype. However, in our opinion, our genetic association data together with the experimental confirmation of altered *MBTPS2* splicing and the previous knowledge on the biological function of MBTPS2 in skin, provide very strong suggestive evidence that *MBTPS2*:c.1437+4T>C does indeed cause the skin and hair coat changes seen in BR1 horses.

In summary, we provided evidence that BR1 in horses is inherited as an X-chromosomal semidominant trait. A positional cloning approach identified the intronic variant c.1437+4T>C and a resulting splice defect in the *MBTPS2* gene as candidate causative mechanism underlying the BR1 phenotype.

## Supplementary Material

Supplemental Material
