# Supplementary material for: An Intronic MBTPS2 Variant Results in a Splicing Defect in Horses with Brindle Coat Texture
Source: G3 (Bethesda). 2016 Jul 22;6(9):2963–70. doi: 10.1534/g3.116.032433 (PMC5015953; doi:10.1534/g3.116.032433)
Supplement: Supplemental Material [file supp_6_9_2963__index.html]

An Intronic MBTPS2 Variant Results in a Splicing Defect in Horses with Brindle Coat Texture — An Intronic MBTPS2 Variant Results in a Splicing Defect in Horses with Brindle Coat Texture — Supplemental Material 

# An Intronic *MBTPS2* Variant Results in a Splicing Defect in Horses with Brindle Coat Texture

## Supplemental Material for Murgiano, *et al*, 2016

**Files in this Data Supplement:**

- Figure S1 - Sanger cDNA sequencing data of *MBTPS2* transcripts. (.pdf, 162 KB)
- File S1 - Methodology for the Haplotype Analyses. (.pdf, 312 KB)
- Table S1 - Genes annotated in the critical interval, chrX:13,601,933-18,711,357. The annotation was retrieved from NCBI Mapviewer and relates to the EquCab 2 assembly. (.xlsx, 16 KB)
- Table S2 - Brindle-associated variants and snpEFF predictions in the critical interval (EquCab 2 assembly). The table contains 61 variants, which were present in heterozygous state in 4 brindle horses and homozygous wildtype in 60 control horses. Note that some variants are listed several times, if they have multiple predicted effects. (.xlsx, 17 KB)
- Table S3 - Control horses from other breeds. (.xlsx, 13 KB)
- Table S4 - Sample designations and breed information on 64 horses with genome sequences. (.xlsx, 16 KB)
