# Supplementary material for: An Intronic MBTPS2 Variant Results in a Splicing Defect in Horses with Brindle Coat Texture
Source: G3 (Bethesda). 2016 Jul 22;6(9):2963–70. doi: 10.1534/g3.116.032433 (PMC5015953; doi:10.1534/g3.116.032433)
Supplement: Supplemental Material [file supp_g3.116.032433_FigureS1.pdf]

A ***MBTPS2*, coding sequence**

```
   1 atgattccgg tgtcgctggt ggtggtggtg gtgggcggct ggactgccgt ctacctgacc
  61 gacttggtgc tgaagtcatc agtgtatttt aagcattctt atgaagactg gctggaaaac
 121 aacggattga gcatttcccc ttttcacatc agatggcaaa ctgctatttt caatcgtgcc
 181 ttttacagtt ggggacggcg gaaagcgagg atgctttacc agtggttcaa ttttggaatg
 241 gtgtttggcg taattgccat gttcagctct tttttcctcc tggggaaaac gctgatgcag
 301 actttagcac aaatgatggc tgattctccc acctcttatt cttcccctc ttcttcctct
 361 tcttcctcct cctcttcttc ctcctcctct tcttcctctt cctcttcttc acttcacaat
 421 gaacaggtgc tacaagttgt ggttcctggt ataaatttac cggtcaacca actgacctat
 481 ttcttcgctg cagtcctcat tagtggtgtt gtacatgaaa ttggacatgg gatagcagct
 541 attagggaac aagtacgatt taatggcttt gggatttttc tcttcattat ttatcctgga
 601 gcatttgttg atctgttcac cactcatttg caacttatat caccagtcca gcagctaagg
 661 atattttgtg caggtatctg gcataatttc gtccttgcac tcctgggtat tttagctctt
 721 gttctgctcc ctgtaattct cttgccattt tactacactg gagtcggggt gcttatcact
 781 gaagttgctg aggactcacc tgccattgga cccagaggcc tttttgtggg agaccttgtc
 841 acccatctac aggattgtcc tgttactaac gtgcaggatt ggaatgaatg tctagatacc
 901 atcgcctatg agccccaaat tggttactgt ataagtgcgt caactttaca gcagttgagc
 961 ttcccagtta gagcatacaa acggctagat ggttcgactg aatgctgtaa caatcacagc
1021 cttacagatg tgtgcttttc ctacagaaat aattttaata agcgtttgca tacatgtcta
1081 cctgctcgga aagcagttga agccactcaa gtttgtagaa ccaataaaga ttgtaaaaaa
1141 agctcaagtt caagtttctg tataattcct tctttggaaa ctcatactcg cttaataaaa
1201 gtgaagcacc cgcctcaaat cgatatgttg tacgtaggac atccactaca tcttcactat
1261 acagtgagta tcactagttt tatcccacgt ttcaactttc taagcataga tctgcctgtg
1321 gttgtggaga catttgtcaa gtacctgatt tccctctcag gagctctggc tattgttaat
1381 gcagtgccct gctttgcttt ggatggacag tggattttaa actctttcct ggatgccacc
1441 cttacctcgg tgattggaga caatgatgtc aaagatctga taggattttt tatcttgctt
1501 ggtggcagta tacttttggc tgctaatgtg accctgggac tctggatggt tacagcacgg
1561 taa
```

B **Control**

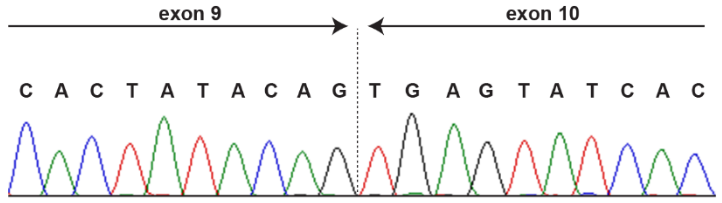

C **Brindle (BR1)**

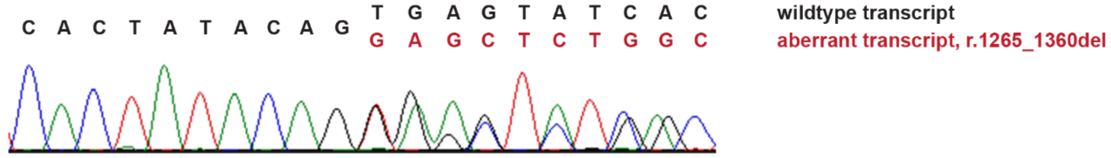

Figure S1 Sanger cDNA sequencing data of *MBTPS2* transcripts. (A) Equine *MBTPS2* reference sequence, derived from Genbank accession XM_005614038.2. The 96 nucleotides missing in the aberrant transcript from brindle horses are underlined. (B) Electropherogram of a control horse, which was homozygous for the wildtype allele. (C) Electropherogram of a brindle mare, heterozygous for the *MBTPS2*:c.1437+4T>C variant. Overlapping signals from two different transcripts can be seen after the end of exon 9. The Sanger sequences were obtained using the forward PCR primer on the RT-PCR products shown in Figure 6 of the main manuscript.
