# Supplementary material for: An Intronic MBTPS2 Variant Results in a Splicing Defect in Horses with Brindle Coat Texture
Source: G3 (Bethesda). 2016 Jul 22;6(9):2963–70. doi: 10.1534/g3.116.032433 (PMC5015953; doi:10.1534/g3.116.032433)
Supplement: Supplemental Material [file supp_g3.116.032433_FileS1.pdf]

Supplemental File 1. Methodology for the Haplotype Analyses

We used LINKPHASE3 (Druet and Georges, 2015) to reconstruct haplotypes in the genotyped pedigree based on Mendelian segregation rules and linkage information. When a parent-offspring pair is genotyped, the program also provides the probability Φ_{ijk} that the offspring i inherited the paternal haplotype of the parent j (1 = sire / 2 = dam) at position k (the probability is 1- Φ_{ijk} for the maternal haplotype). Using these, we can compute the probability, for all genotyped individuals in the pedigree, to inherit either paternal or maternal haplotype of a founder F, by recursively using the following equation from the founder to the individuals from the most recent generation:

$$\mathrm{P}\left(\mathrm{H}_{\mathrm{i,j,k}} = \mathrm{H}_{\mathrm{F,l,k}}\right) = \phi_{\mathrm{ijk}}\mathrm{P}\left(\mathrm{H}_{\mathrm{p_{ij},1,k}} = \mathrm{H}_{\mathrm{F,l,k}}\right) + (1-\phi_{\mathrm{ijk}})\mathrm{P}\left(\mathrm{H}_{\mathrm{p_{ij},2,k}} = \mathrm{H}_{\mathrm{F,l,k}}\right)$$

where $H_{i,j,k}$ represents the paternal ($j=1$) or maternal ($j=2$) haplotype from individual i at position k, and p_{ij} is the sire ($j=1$) or the dam ($j=2$) of individual i. The probability that individual i inherited at least one copy of haplotype l from founder F at position k is:

$$\mathrm{P}\left(\mathrm{A}_{\mathrm{i,k}} \leftarrow \mathrm{H}_{\mathrm{F,l,k}}\right) = \mathrm{P}\left(\mathrm{H}_{\mathrm{i,1,k}} = \mathrm{H}_{\mathrm{F,l,k}}\right) + \mathrm{P}\left(\mathrm{H}_{\mathrm{i,2,k}} = \mathrm{H}_{\mathrm{F,l,k}}\right) - \mathrm{P}\left(\mathrm{H}_{\mathrm{i,1,k}} = \mathrm{H}_{\mathrm{F,l,k}}\right)\mathrm{P}\left(\mathrm{H}_{\mathrm{i,2,k}} = \mathrm{H}_{\mathrm{F,l,k}}\right)$$

The probability that individual i did not inherit a copy of haplotype l from founder F at position k is simply:

$$\mathrm{P}\left(\mathrm{A}_{\mathrm{i,k}} \nleftarrow \mathrm{H}_{\mathrm{F,l,k}}\right) = 1 - \mathrm{P}\left(\mathrm{A}_{\mathrm{i,k}} \leftarrow \mathrm{H}_{\mathrm{F,l,k}}\right)$$

The probability to observe the disease if animal *i* inherited a copy of haplotype *l* from founder *F* at position *k* is 1.00 if that haplotype carriers the causative variant ($H_{F,l,k} \equiv *$),

$$P(Y_i = D | A_{i,k} \leftarrow H_{F,l,k}, H_{F,l,k} \equiv *) = 1$$

Similarly, the probability to observe a normal phenotype is null with a similar transmission pattern.

$$P(Y_i = N | A_{i,k} \leftarrow H_{F,l,k}, H_{F,l,k} \equiv *) = 0$$

If animal *i* did not inherit a copy of haplotype *l* from *F* at position *k*, we obtain the opposite (complementary) probabilities:

$$P(Y_i = D | A_{i,k} \nleftarrow H_{F,l,k}, H_{F,l,k} \equiv *) = 0$$
$$P(Y_i = N | A_{i,k} \nleftarrow H_{F,l,k}, H_{F,l,k} \equiv *) = 1$$

The probability $P(Y_i | H_{F,l,k} \equiv *)$ to observe phenotype *i* if haplotype *l* from founder *F* carriers the causative variant is:

$$P(A_{i,k} \leftarrow H_{F,l,k})P(Y_i | A_{i,k} \leftarrow H_{F,l,k}, H_{F,l,k} = *) + P(A_{i,k} \nleftarrow H_{F,l,k})P(Y_i | A_{i,k} \nleftarrow H_{F,l,k}, H_{F,l,k} = *)$$

To test whether haplotype *l* from founder *F* carries the causative variant, we compute the likelihood to observe the vector of phenotypes **Y** under that assumption:

$$L(Y|\ H_{F,l,k} \equiv *) = \prod_{i=1}^{n} P(Y_i|\ H_{F,l,k} \equiv *)$$

This likelihood is null if 1) any of the affected individuals has a null probability to inherit a copy of the founder haplotype at position *k* or 2) we are certain that at least one of the unaffected inherited the haplotype.

In regard of the sexual chromosomes, we modified LINPKHASE3 with the following rules. First, the pseudo-autosomal region (PAR) was treated as any other autosome. The PAR boundary was set between two markers positioned 1,359,986 and 1,429,389 (EquCab 2 assembly); this was based on homozygosity/heterozygosity patterns in males and on number of observed opposite homozygotes in sire/sons pairs (we expect no opposite homozygotes in the PAR because the sire transmits one allele to the sons; on the X-specific region, a son's genotype depends only on the allele received from the dam and opposite homozygotes are common).

For the X-specific region, females were considered diploid and males haploid. Transmission from mothers to offspring followed the same rules as autosomes, whereas males transmitted their unique haplotype to daughters, and nothing to sons.
